# Nervous System Injury in Response to Contact With Environmental, Engineered and Planetary Micro- and Nano-Sized Particles

**DOI:** 10.3389/fphys.2018.00728

**Published:** 2018-06-26

**Authors:** Tatiana Borisova

**Affiliations:** Department of Neurochemistry, Palladin Institute of Biochemistry, National Academy of Sciences of Ukraine, Kiev, Ukraine

**Keywords:** micro- and nano-sized particles, air pollution particulate matter, particle biocorona, nervous system, neurosafety

## Abstract

Nerve cells take a special place among other cells in organisms because of their unique function mechanism. The plasma membrane of nerve cells from the one hand performs a classical barrier function, thereby being foremost targeted during contact with micro- and nano-sized particles, and from the other hand it is very intensively involved in nerve signal transmission, i.e., depolarization-induced calcium-dependent compound exocytosis realized via vesicle fusion following by their retrieval and calcium-independent permanent neurotransmitter turnover via plasma membrane neurotransmitter transporters that utilize Na^+^/K^+^ electrochemical gradient as a driving force. Worldwide traveling air pollution particulate matter is now considered as a possible trigger factor for the development of a variety of neuropathologies. Micro- and nano-sized particles can reach the central nervous system during inhalation avoiding the blood–brain barrier, thereby making synaptic neurotransmission extremely sensitive to their influence. Neurosafety of environmental, engineered and planetary particles is difficult to predict because they possess other features as compared to bulk materials from which the particles are composed of. The capability of the particles to absorb heavy metals and organic neurotoxic molecules from the environment, and moreover, spontaneously interact with proteins and lipids in organisms and form biomolecular corona can considerably change the particles‘ features. The absorption capability occasionally makes them worldwide traveling particulate carriers for delivery of environmental neurotoxic compounds to the brain. Discrepancy of the experimental data on neurotoxicity assessment of micro- and nano-sized particles can be associated with a variability of systems, in which neurotoxicity was analyzed and where protein components of the incubation media forming particle biocorona can significantly distort and even eliminate factual particle effects. Specific synaptic mechanisms potentially targeted by environmental, engineered and planetary particles, general principles of particle neurosafety and its failure were discussed. Particle neurotoxic potential depends on their composition, size, shape, surface properties, stability in organisms and environment, capability to absorb neurotoxic compounds, form dust and interrelate with different biomolecules. Changes in these parameters can break primary particle neurosafety.

## Rationale

Growing number of patients with neurological and neurodegenerative disorders and diseases, etiology of which is far from being clear, is burden of modern society. An increasing amount of environmental pollutants, especially particulate matter components of air pollution, can be responsible for the development of such pathologies (Eisen et al., 2011; Block et al., 2012; Karmakar et al., 2014). Importantly, particulate airborne pollutants appeared in one region travel across state boundaries, oceans and continents and so disperse globally (Jiang et al., 2015; Zhang et al., 2017). Up to 11% of black carbon air pollution components detected in the western United States were originated from Chinese manufacturer emissions (Lin J. et al., 2014; Landrigan et al., 2018). The main sources of air pollution particulate matter include black carbon from fuel exhaust, wood and coal combustion, plastic and garbage burning in undeveloped countries. Beside that air pollution particulate matter can contain engineered particles used in agriculture nanotechnologies and also possible deposits from industry and medical nanotechnologies because the latter are very perspective, and so enormously growing modern scientific fields. Both household and ambient air pollution are considered separately, whereas they often co-exist and comprise similar pollutants (Chafe et al., 2014; Pruss-Ustun et al., 2016; Landrigan et al., 2018).

Tons of cosmic dust from meteorites, comets and other objects of the solar system fall on the Earth daily. Also, beside problems associated with altered gravity, radiation, etc, threat to the nervous system during long-term space flight and planet colonization can come from planetary and interstellar dust exposure.

**FIGURE 1:**
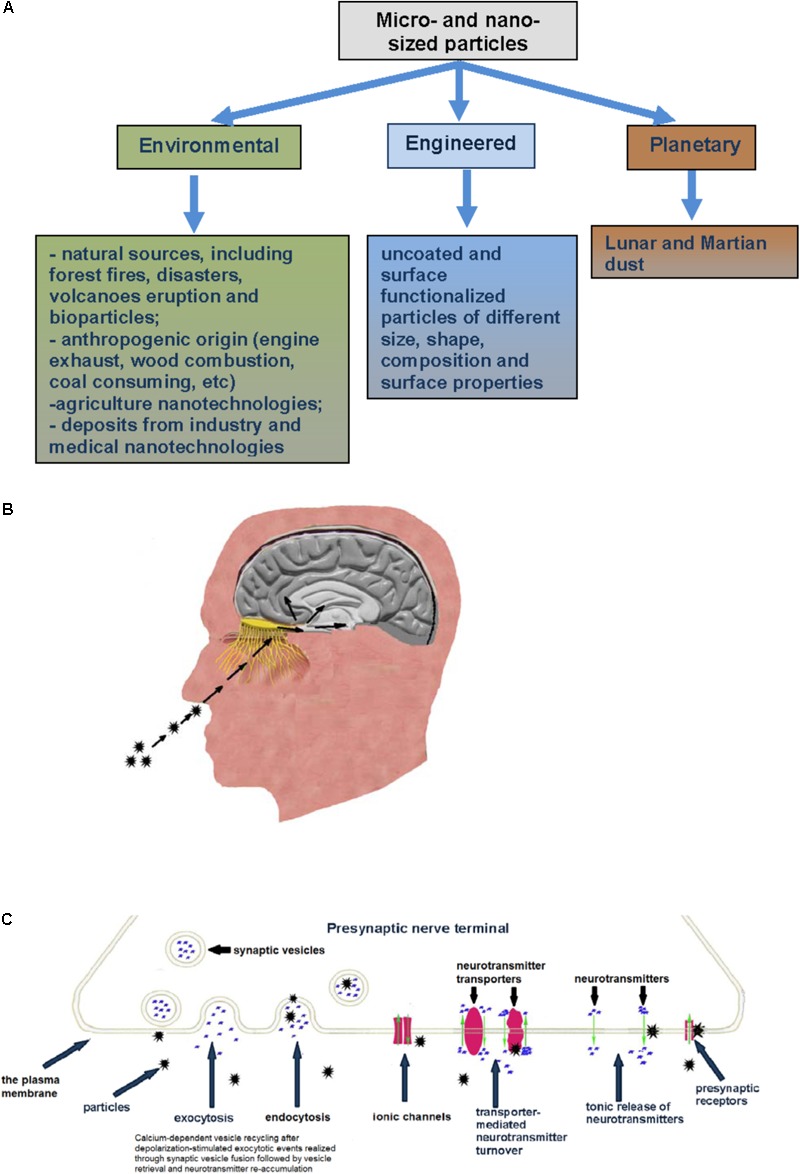
**(A)** Types of micro- and nano-sized particles reviewed in this paper. **(B)** Inhaled particles deposited on the rat olfactory mucosa can translocate along olfactory nerve sensory axons, circumvent the BBB and target the CNS. **(C)** Specific mechanisms and membrane proteins in nerve cells as potential targets for micro- and nano-sized particle influence (see explanation in the text).

Novel experimental data demonstrate that the central nervous system (CNS) is extremely sensitive to the influence of micro- and nano-sized particles (Landrigan et al., 2018). Currently, the methodology for neurotoxicity risk assessment of micro- and nano-sized particles of environmental, engineered and space origin (**Figure 1A**) is quite uncertain and still remains almost undeveloped. From the one hand, this is because of both the relatively recent recognition of nanotechnology and also new consideration regarding involvement of air pollution particulate matter in the etiology of different neuropathologies, and from the other hand – because of enormous micro- and nano-sized particle diversity. Precise prognosis regarding neurotoxicity of the particles cannot be made even when they originate from well-characterized materials. It is so because bulk materials change their features when they act in a micro- or nano-sized form. Then, the particles change their properties during interference with biological objects, entrance to living organisms and interaction with proteins and lipids that spontaneously form biocorona at the particle surface. Also, properties of micro- and nano-sized particles can be changed by spontaneous absorption of a variety of neurotoxic compounds from the environmental surrounding.

## Routes of CNS Access for Micro- and Nano-Sized Particles

Normal brain functioning requires maintenance of precise homeostasis of ions and molecules between the blood and brain by tight control and regulation of their transportation. It is realizing through a unique blood–brain barrier (BBB), which comprises a highly specialized endothelial cells adjoined by tight cell-to-cell junction forming a seal between opposing endothelial membranes. This barrier is characterized by specific transport mechanisms, a low pinocytosis rate and a weak permeability with restriction of paracellular solute diffusion from the blood (Reese and Karnovsky, 1967; Lochhead and Thorne, 2012). Specific transporters transfer essential nutrients to the brain, whereas small lipophilic molecules can cross the BBB by passive diffusion.

Numerous neuropathologies, e.g., stroke, multiple sclerosis, brain tumors, Parkinson’s and Alzheimer’s diseases and neuroinflammation are accompanied by BBB dysfunction (Luissint et al., 2012). Disturbance of the BBB permeability can result in increased CNS accessibility for different types of micro- and nano-sized particles, thereby making such patients more suffer from particle exposure.

The CNS can be classified as a secondary exposed tissue during organism contact with micro- and nano-sized particles as it is not subjected to direct interaction with them. In the circulation, nano-sized particles can switch on cascade mechanisms disrupting the endothelial cell tight junctions in the BBB and change the membrane permeability. It has been demonstrated that intravenously, intraperitoneally and intracerebrally administrated gold, cuprum and aluminum nano-sized particles disrupt the BBB (Karmakar et al., 2014). Nano-sized particles can stimulate vesicular transport, thereby getting CNS access. Nanoparticle hydrophobicity can increase BBB penetration. However, additional research is necessary for complete understanding of the mechanisms of particle translocation from the blood to the brain via the BBB (Karmakar et al., 2014).

Importantly, micro- and nano-sized particles can reach the CNS during inhalation and are deposited in nasal, tracheobronchial and alveolar regions in mammalian organisms (Oberdörster et al., 2004; Kao et al., 2012; Wang et al., 2016). A 12-week inhalation experiment with titanium dioxide nano- and micro-sized particles of the same crystalline structure resulted in a similar mass deposition in the lower respiratory tract of rats (Oberdörster et al., 1994, 2005; Oberdörster, 2000). The surface area of the lung is ∼80–140 m^2^ with thin cellular layer of ∼0.1–0.2 microm (Scheuch et al., 2006).

The precise mechanisms by which the particles can travel from the nasal epithelium to the CNS have not been completely understood. The nasal cavity of humans lengthens ∼12–14 cm and is of a huge absorptive surface (∼160 cm^2^), which also aids in filtering inspired air (Harkema et al., 2006; Lochhead and Thorne, 2012). Deposition of micro- and nano-sized particles on the olfactory mucosa of the nasopharyngeal region of the respiratory tract has been confirmed experimentally. Literature data have demonstrated that nasal delivery can be effective because mucosal sites are characterized by relatively high permeability and nasal regions of mammalian organisms can uptake nano-sized particles. Then the particles are subsequently translocated along olfactory nerve sensory axons (Kao et al., 2012), circumvent the BBB and target the CNS. One fifth of nano-sized particles deposited on the rat olfactory mucosa can move to the brain olfactory bulb (Oberdörster et al., 2004) (**Table 1** and **Figure 1B**). Intranasally instilled nano-sized maghemite particles were transported into the brain via the olfactory route (Wang et al., 2007; Karmakar et al., 2014). Titanium dioxide nano-sized particles were found in the mice brain after particle administration (Takeda et al., 2009). Chronic particle inhalation can trigger the mediator release to circulation (Kreyling et al., 2013). Intranasally instilled silver-coated colloidal gold particles in monkeys moved in the axons of the olfactory nerve to the olfactory bulbs (de Lorenzo, 1970; Oberdörster et al., 2005). Manganese, cadmium telluride and cobalt nanomaterials can translocate from exposed olfactory epithelium via olfactory neurons to the brain (Persson et al., 2003; Elder et al., 2006; Zhang et al., 2007). After that the particles can be transported from the sites of their initial brain entry to other sites in the CNS (Lochhead and Thorne, 2012). The main sites for deposition of nano-sized magnetite particles after intranasal instillation were the olfactory bulb, striatum and hippocampus. In 7 days after instillation, nano-sized particles (∼80%) were still found in the striatum and ∼50% of them were registered after 14 days in the striatum and hippocampus (Karmakar et al., 2014). Silver nano-sized particles during inhalation exposure can translocate to the brain using the nasopharyngeal system (Oberdörster et al., 2004; Karmakar et al., 2014). Beside entry into the cranial compartment via olfactory nerve bundles, particle uptake after nasal delivery can be through olfactory blood vessel absorption flowing by entry into the general circulation and olfactory lymphatic vessel absorption (Lochhead and Thorne, 2012; Chapman et al., 2013). Intranasal administration of proteins using nano-sized particles was considered as promising medical approach especially for mucosal vaccines (Bernocchi et al., 2016). Similar pathway was demonstrated for intranasal administration of [^125^I]-labeled proteins in rats and monkeys, where protein delivery was performed along the olfactory and trigeminal nerves in the nasal epithelium to the olfactory bulb and brainstem, respectively, and then further dispersed in the CNS (Thorne et al., 2004; Thorne et al., 2008). Particle hydrophobicity can increase efficiency of their brain delivery during intranasal exposure. It was shown that antidepressant drug agomelatine solid lipid nanoparticles more effectively targeted the brain during the intranasal administration as compared to the intravenous route (Fatouh et al., 2017).

**Table 1:** A chronologic table of key studies on intranasal administration of micro- and nano-sized particles.

Types of micro- and nano-sized particles	Reference
Silver-coated colloidal gold particles	de Lorenzo, 1970; Oberdörster et al., 2005
Titanium dioxide nano- and micro-sized particles of the same crystalline structure	Oberdörster et al., 1994, 2005; Oberdörster, 2000
Cobalt-containing dust	Persson et al., 2003
Ultrafine elemental (13)C particles from [(13)C] graphite rods	Oberdörster et al., 2004
Airborne nano-sized particles	Oberdörster et al., 2005
Ultrafine manganese oxide particles	Elder et al., 2006
Cadmium telluride nano-sized particles	Zhang et al., 2007
Micro- and nano-sized maghemite particles	Wang et al., 2007
Titanium dioxide nano-sized particles	Takeda et al., 2009
Zink oxide nano-sized particles	Kao et al., 2012
Biopersistent nano- and micro-sized particles	Kreyling et al., 2013
Cationic porous nano-sized particles loaded with protein	Bernocchi et al., 2016
Antidepressant drug agomelatine solid lipid nano-sized particles	Fatouh et al., 2017

In environmental studies, pathway through olfactory nerve can serve for airborne nano-sized particles as an entry portal to the human CNS (Karmakar et al., 2014).

## Specific Mechanisms in Nerve Cells that Can Be Targeted by Micro- and Nano-Sized Particles

Human organism and the CNS in particular were subjected during evolution to influence of micro- and nano-sized particulate matter, for instance natural mineral particles, carbon-containing particles from forest fire storms, space, etc. Growing application of engineered particles increases their presence in the natural environment and various food chains (Goswami et al., 2017). Permanent exposure to carbon-containing particles from intensive anthropogenic activity, and civilization achievement associated with incomplete wood combustion, diesel exhaust, industry deposits, food cooking can be accompanied with health problems. The relevant physiological mechanisms can be not fully adapted to protect the CNS from modern particle influence.

Despite the CNS is effectively protected by the BBB from the action of different substances in the molecular form, we can conclude from the previous subsection that this barrier does not strand against evolutionary new challenge of micro- and nano-particle exposure. This secure barrier is mainly unsafe and does not completely protect the CNS functioning during particle exposure associated primarily with modern and industrial anthropogenic activity (**Figure 1B**).

Capability of micro- and nano-sized particles to overcome the BBB from the one hand can bring crucial problems in environmental neurotoxicology, but from the other hand this particle feature is considered promising in nanoneurotechnology for drug delivery, imaging, treatment and neurotheranostics.

Nerve cells in organisms take an exceptional place among other cells because they possess unique physiological function mechanism. The plasma membrane of nerve cells is intensively involved in rapid calcium-dependent vesicle recycling after depolarization-stimulated compound exocytotic events. It is realized through synaptic vesicle fusion followed by vesicle retrieval and neurotransmitter re-accumulation. This mechanism is important to make synaptic vesicles ready to new exocytotic events. In this context, nano-sized particles can be uptaken by synaptic vesicles during vesicle recycling, thereby changing their normal functioning, occupying neurotransmitter storage area inside of them and attenuating exocytotic release efficiency.

Between periodically occurred exocytotic events, the main activity is underscored by maintenance of the proper ambient level of fast neurotransmitters, for example glutamate, GABA, glycine, aspartate, through their permanent turnover across the plasma membrane driven by Na^+^-dependent neurotransmitter transporters (Borisova, 2016; Borisova and Borysov, 2016). The functioning of these transporters strongly depends on physical and chemical properties of the membrane (Borisova, 2013) and any changes in the membrane properties triggered by micro- and nano-sized particles can significantly disturb transporter functioning. Possible particle-induced dissipation of plasma membrane ionic gradients disturbs the nerve cell membrane potential that is in turn a driving force for transporter-mediated neurotransmitter uptake. Particle-induced membrane depolarization can also affect following exocytotic events. Tonic unstimulated release from presynaptic nerve terminals between episodes of exocytosis is an important constituent involved in maintenance of definite ambient level of the neurotransmitters and release/uptake balance. Any changes in membrane permeability resulted from destructive contact with micro- and nano-sized particles can significantly impact tonic neurotransmitter release. It is clear that particle-induced alteration of plasma membrane properties can also affect ionotropic (and metabotropic) neurotransmitter receptor functioning at the postsynaptic site. Postsynaptic signaling can be also influenced by particle-evoked changes in the neurotransmitter concentration in the synaptic cleft and strength of exocytotic signal (**Figure 1C**).

So, from the one hand the plasma membrane of nerve cells is involved extremely intensively in nerve cell functioning and signal transmission, and from the other hand it simultaneously performs the classical barrier and compartmentalization function. The plasma membrane is a first target for micro- and nano-sized particle influence when they reach and contact with nerve cells. In this context, nerve signal transmission and the CNS functioning in a whole can be extremely susceptible and sensitive to particle impact. Importantly, changes in plasma membrane properties, e.g., in the cholesterol level, can influence its barrier function regarding resistance to micro- and nano-sized particle exposure.

## General Aspects of Micro- and Nano-Sized Particle Neurotoxicity

Nowadays, general principles and methods for neurotoxicity risk assessment of environmental, engineered and planetary micro- and nano-sized particles are quite uncertain and still undeveloped. The neurotoxic potential is difficult to predict because the particles possess different features in comparison with bulk precursor materials from which they are derived from. Well-characterized compounds change their properties when they are transformed into the micro- and nano-sized forms. For engineered particles, it occurs during their synthesis and surface functionalization and for environmental ones – through exposure of bulk materials to environmental factors and during anthropogenic activity. The particles can change their properties during further interaction with neuroactive environmental pollutants and then with proteins, lipids, nucleic acids, etc., in living organisms being coated with biocorona. This unpredictable path can haphazardly and tremendously modify particle features making almost impossible integrated neurotoxicity risk prognosis.

The particle size is very important parameter that can significantly contribute to their neurotoxic potential and determine their interaction with cells and cellular internalization. Nanoparticles, the size of which is less than 5 nm, are considered to be the most hazardous because of a nuclear access possibility and an extremely large ratio of surface parameters related to volume ones. Nanoparticles, the size of which is higher than 40 nm demonstrated a reducing internalization efficiency and cytotoxic effects (Soenen et al., 2011, 2015). Nevertheless, the suggestion on significantly higher acute toxicity of smaller nanoparticles in comparison with larger ones is still doubtful and should be further investigated.

Features of particle surface area can also considerably influence their neurotoxicity. Carbon nanodots synthesized from different precursors, i.e., sulfur-containing and sulfur-free ones, thiourea and β-alanine, respectively, possessed diverse surface characteristics, and therefore displayed a diverse level of efficiency; however, their effects were unidirectional in brain nerve terminals (Borisova et al., 2017). Nanodiamonds obtained using different synthesis protocol had diverse surface shape and charge characteristics, and thereby varied in intensity of exhibited neurotoxic effects (Pozdnyakova et al., 2016). Cationic nanoparticles exerted the highest cytotoxic effects were less stable (Geys et al., 2008; Singh et al., 2009; Soenen et al., 2011), caused more significant disruption of plasma membrane integrity, and mitochondrial and lysosomal damages than anionic ones (Fröhlich, 2012). Positive surface charge reduction can decrease cellular internalization (Soenen et al., 2007, 2011). Cerium oxide nanoparticles with a functionalized surface and a positive and neutral charge were internalized to normal cell lines, whereas negatively charged ones entered presumably the cancer cell lines (Asati et al., 2010). Neutral carbon dots did not influence cell morphology and did not induce cell cycle abnormalities, negatively charged ones arrested the G2/M phase of the cell cycle, stimulated proliferation and led to higher oxidative stress. Whereas positively charged carbon dots were the most cytotoxic, entered the cell nucleus and induced significant changes in G0/G1 phase of cell cycle (Havrdova et al., 2016).

The shape effects were demonstrated using gold nanorods that were less toxic than the spherical nanoparticles (Qiu et al., 2010; Aaron et al., 2011; Soenen et al., 2011; Tarantola et al., 2011). Volcanic dust particles with sharp angles can somehow interact with the nerve terminal plasma membrane, change its surface properties and enhance L-[^14^C]glutamate binding, whereas volcanic dust particles of spherical form did not show this effect (Pozdnyakova et al., 2017). Particle size, shape, composition and surface properties are critical for their uptake and pharmacokinetic (Hoshyar et al., 2016).

Capability of micro- and nano-sized particles to release neurotoxic metals, components of core and surface area, and other neuroactive compounds included in their structure can significantly contribute to particle neurotoxic potential. It was shown that the dissolution of metal ions from metal oxide nanomaterials made a considerable input to their neurotoxicity (Tallkvist et al., 2002; Karmakar et al., 2014). A significant increase in iron content in almost all brain regions, the olfactory bulb, hippocampus, cerebral cortex, cerebellum and the brainstem was detected after a single intranasal administration of maghemite nanoparticles of 21 nm in size (Wang et al., 2008). Neurotoxic effects from airborne aluminum inhalation indicated that the subclinical neurological symptoms and low phospholipid-binding Clara cell protein CC16 level could be associated with an internalization of aluminum ions by the lung epithelium and BBB penetration (Halatek et al., 2008). Toxicity of zinc oxide nanoparticles was caused by Zn^2+^ dissolved outside or inside the cells in culture (Deng X. et al., 2009).

Modification of the size, shape, composition, surface properties (including environmental transformation and biocorona formation, see below), stability in organisms (including release of toxic substances), dust-forming capability of micro- and nano-sized particles can unexpectedly convert them from non-neurotoxic to potentially neurotoxic ones and vice versa. So, the exact effects of abovementioned factors on particle neurotoxicity need thorough understanding.

## CNS Hazard Associated with Exposure to Environmental Micro- and Nano-Sized Particles

The amount of death associated with ambient air pollution originated from modern industrial and urban development is growing. The total number of death attributable to air pollution particulate matter the size of which is 2.5 μm (PM2.5) increased by 20% from 3.5 million in 1990 to 4.2 million in 2015. A mortality prognosis associated with ambient PM2.5 air pollution has revealed that the number of death will increase over the next three decades by more than 50% upto 6.6 million in 2050 (Pope et al., 2011; Lelieveld et al., 2015; The Lancet, 2016; Landrigan et al., 2018).

Essential sources of air pollution carbon-containing particles are coal consuming technologies, operation of diesel engines, wood combustion for residential heating in the cold season and other areas of anthropogenic activity and natural disasters, that is, forest fires, oil, biomass and garbage burning. Also, tons of the particles including carbon-containing ones fall on the Earth daily (Garai et al., 2006; Pizzarello and Shock, 2010). Wood combustion and biomass burning contribution to organic carbon at European sites varies between 30 and 75% (Gelencsér et al., 2007; Szidat et al., 2007; Gilardoni et al., 2016). The use of wood for residential heating is spreading in developed countries and so the number of the studies related to health outcomes is growing (Fullera et al., 2014; Fuzzi et al., 2015). Carbon-containing particles due to their small size can form aerosols that can be spread by wind to large distances. Chemical and physical features of wood-combusting particles can vary considerably in dependence on the burning conditions, technologies, applied tools and biomass types. Possessing highly adhesive surface, aerosol carbon-containing particles can bind different molecules from the surrounding that makes their surface charged and oxidized. Becoming water soluble carbon-containing particles can also contaminate water. Water soluble organic carbon emitted by heavy-duty engines showed high oxidative potential (Biswas et al., 2009; Cheung et al., 2009).

PM2.5 air pollution was demonstrated to be associated with a wide range of diseases, among which cardiovascular and pulmonary diseases are in the first place and are well-characterized showing the strongest causal PM2.5 associations. Emerging evidences promise that a causal association may exist between PM2.5 pollution and lowered cognitive function, autism and attention-deficit or hyperactivity disorder in children, neurodegenerative disease, including dementia in adults and stroke (Landrigan et al., 2018). It has been shown in modeling experiments that carbon nanoparticles synthesized from carbohydrates possessed neurotoxic effects (Borisova et al., 2015, 2017). So, the presence of abandoned carbon-containing particles in aerosols poses a neurotoxicity risk especially during natural disasters. Air pollution-mediated changes in the immune system simultaneously can alter the brain functioning through the production of circulating proinflammatory mediators (Block et al., 2012). Nanoparticle chemical herbicides are also considered to belong to pollutants, which effects on human health and the CNS in particular are only launching to be analyzed (Landrigan et al., 2018). It has been indicated in the previous subsection that the lesser the size of the particles is, the more significant health hazard is expected. In this context, not only PM2.5, but also potentially ultra dangerous PM1 air pollution is starting to be monitored.

The feasible brain health hazard from environment-derived micro- and nano-sized particles was demonstrated in recent study, where the magnetite nanoparticles were found in the human brain (Maher et al., 2016). Growing engineered particle production increases their presence in the environment. Surface functionalized particles used in different technologies being released to the environment can lose their coating and after that can accordingly change their neurosafety. In this context, neurotoxicity risk assessment of engineered particles released to the environment needs to be performed taking into account their further chemical and biodegradation processes.

## A Comparative Analysis of Neurotoxic Potential of Engineered Micro- and Nano-Sized Particles

Surface functionalized nanoparticles for targeted CNS drug delivery, neuroimaging, neurological disease treatment and neurotheranostics are in the main stream of modern research and development in the field of nanotechnology and nanomedicine. A lot of studies show a great progress in this valuable and prospective field. However, literature data examination reveals a lack of systematic analysis of neurosafety of different types of engineered micro- and nano-sized particles. This fact underscores importance and necessity of parallel comparative assessment of particle neuroactive features performed using similar methodological approaches.

Titanium oxide nanoparticles have been used extensively and broadly. Their accumulation in the brain influenced metabolism and transport of neurotransmitters norepinephrine and serotonin (Wang et al., 2007; Hu et al., 2010; Ma et al., 2010). Animals treated with titanium oxide nanoparticles demonstrated increasing cytokine levels that indicated inflammatory effects in the brain (Wang et al., 2008; Shin et al., 2010). Prenatal exposure of mice to titanium oxide nanoparticles resulted in the augmented levels of homovanillic acid, dopamine, 3,4-dihydroxyphenylacetic acid and 3-methoxytyramina hydrochloride in the prefrontal cortex and neostriatum (Takahashi et al., 2010).

In isolated rat hippocampal CA3 pyramidal neurons, zink oxide nanoparticles induced depolarization, voltage-gated sodium channel activation, release of glutamate, and so neuronal excitability. Zink oxide nanoparticles can deplete intracellular K^+^ increasing its efflux and induced neuronal apoptosis (Zhao et al., 2009; Karmakar et al., 2014).

Silver nanoparticles can provoke inflammation, the BBB integrity disturbance, astrocyte swelling and neuronal degeneration (Tang et al., 2009; Trickler et al., 2010; Karmakar et al., 2014). Intranasally administered silver nanoparticles impaired hippocampal function in rats (Liu et al., 2012) and after intravenous injection they decreased motor activity (Zhang et al., 2013). In experiments *in vitro* and *in vivo* with rat cerebellar granule cells, it has been shown that silver nanoparticles decreased primary neuronal cell survival through apoptosis coupled to oxidative stress (Yin et al., 2013; Karmakar et al., 2014).

**FIGURE 2:**
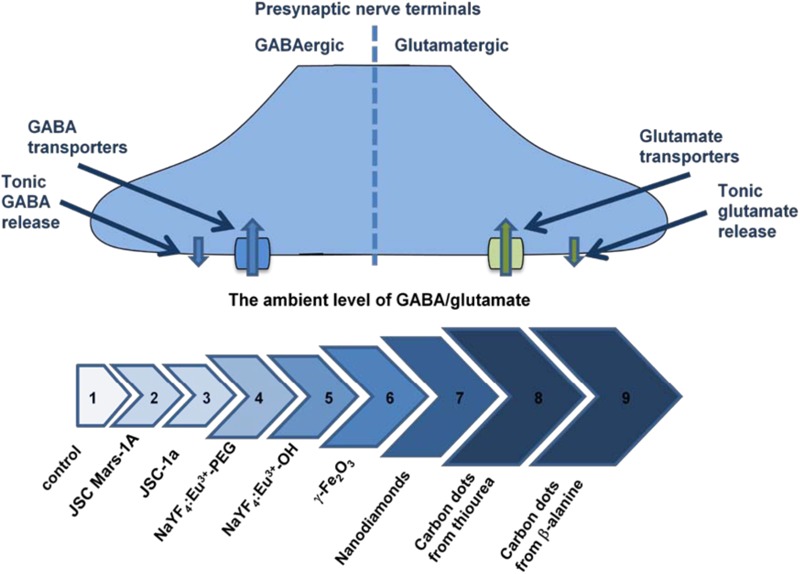
The ambient level of [^3^H]GABA and L-[^14^C]glutamate in the preparations of nerve terminals in the presence of different types of nanoparticles. Arrows’ line segments: #1– Control (Borisova, 2013); #2 – Volcanic ash particles JSC, Mars-1A, 2.0 mg/ml (Pozdnyakova et al., 2017); #3 – Volcanic ash particles JSC-1a, 2.0 mg/ml (Pozdnyakova et al., 2017); #4 – Nanocrystals NaYF_4_:Eu^3+^-PEG, 7.5 mg/ml (Sojka et al., 2017); #5 – Nanocrystals NaYF_4_:Eu-^3+^OH, 3.5 mg/ml (Sojka et al., 2017); #6 – Maghemite nanoparticles γ-Fe_2_O_3_, 0.75 mg/ml (Horák et al., 2017); #7 – Nanodiamonds, 1.0 mg/ml (Pozdnyakova et al., 2016); #8 – Carbon nanodots synthesized from thiourea, 1.0 mg/ml (Borisova et al., 2017); #9 – Carbon nanodots synthesized from β-alanine, 0.4 mg/ml (Borisova et al., 2015).

Comparative analysis of neurosafety levels of different types of micro- and nano-sized particles was carried out in parallel experiments using similar methodological approach. Unique dynamic balance of inhibition and excitation determines brain functioning of individuals and its disturbance contributes to the pathogenesis of main neurological disorders. Individual ambient concentrations of GABA and glutamate in the synaptic cleft are maintained due to neurotransmitter uptake by the plasma membrane neurotransmitter transporters in nerve terminals (Borisova, 2016; Borisova and Borysov, 2016). Changes in the membrane properties resulted from interaction with the particles can consequently alter the functioning of key membrane proteins involved in synaptic neurotransmission and provoke development of neurological consequences. Several nanoparticle types were analyzed and misbalance of the ambient levels of inhibitory/excitatory neurotransmitters was revealed. The strength of this disturbance increased from volcanic ash particles (Pozdnyakova et al., 2017), NaYF_4_ nanocrystals doped with Eu^3+^ (Sojka et al., 2017), maghemite nanoparticles γ-Fe_2_O_3_ (Horák et al., 2017), nanodiamonds (Pozdnyakova et al., 2016) to carbon nanodots synthesized from thiourea (Borisova et al., 2017) and β-alanine (Borisova et al., 2015) (**Figure 2**). This data has shown that synaptic neurotransmission and inhibitory/excitatory balance are exceptionally susceptible to nanoparticle administration.

## Absorbance of Heavy Metals and Neurotoxic Organic Compounds at the Surface of Micro- and Nano-Sized Particles

Heavy metals can reach living organism in the ionic form and can be transported to the nerve cells through the divalent cation/metal-ion transporters. Incorrect metal ion accumulation was revealed in affected substantia nigra neurons of Parkinson’s disease patients (Hirsch and Faucheux, 1998). Children are predominantly susceptible to lead exposure because of the permeable BBB. Lead poisoning is characterized by several symptoms including headache, irritability, abdominal pain, behavioral troubles and memorizing/learning difficulties. Presynaptic malfunction after lead and cadmium administration resulted from partial dissipation of the synaptic vesicle proton gradient following by a decrease in transporter-mediated uptake and exocytotic release of glutamate (Borisova, 2014).

A serious threat to the CNS from exposure to micro- and nano-sized particles comes from the fact that they can be a particulate carrier for brain delivery of heavy metals and other toxic substances bypassing the BBB. For example, heavy metals at the surface of the particles can enter nerve cells and absorbed components can be released to the cytoplasm disturbing the functioning of the synaptic vesicles. In this context, porous-containing micro- and nano-sized particles having high absorbance capability can bring serious threat to brain health. Nevertheless, potential neurotoxic features are inherent not only to this particle type, but also to a range of the particles that can spontaneously interact with heavy metals and with a wide variety of toxic organic molecules. Maghemite nanoparticles are of a special attention because of their high absorption capability. It was shown that functionalized maghemite nanoparticles removed lead and copper from aqueous media (Guivar et al., 2017). These nanoparticles absorbed lead within minute time interval and so they were suggested for removal of heavy metal from electroplating wastewater (Cheng et al., 2012). Also, maghemite nanoparticles possessed high efficiency toward rapid adsorption of fluoride (Jayarathna et al., 2015) and were able to chemisorb aspartate and glutamate (Sousa et al., 2001).

Health impact of organic molecules together with heavy metals as components of environmental airborne particulate matter is very extensively analyzing and remediation technologies are proposing (Delfino et al., 2010; Fuzzi et al., 2015; Popek et al., 2017). Among organic molecules, a special hazard can come from interaction of micro- and nano-sized particles with (^∗^) polychlorinated biphenyls and (^∗∗^) methyl mercury that themselves caused cognitive impairments especially in children exposed prenatally (Grandjean and Landrigan, 2014), (^∗∗∗^) organophosphate pesticide, (^∗∗∗∗^) chlorpyrifos, which caused microcephaly at birth, delays in brain development and autistic behaviors in children as a result of prenatal exposure (Bouchard et al., 2011; Rauh et al., 2012), and (^∗∗∗∗∗^) polycyclic aromatic hydrocarbons associated with neurodevelopmental delays in children exposed prenatally (Perera et al., 2014; Jedrychowski et al., 2015). Also, the neonicotinoids, a novel class of neurotoxic pesticides that persist for years in soils and dusts, can have negative effects on behavior and health of the bees (Rundlöf et al., 2015; Woodcock et al., 2016). However, very little information is available on their possible effects on human brain health (Jeschke et al., 2011). A large diversity of environmental micro- and nano-sized particles and neurotoxic pollutants requires separate assessment of interaction mechanisms for each complex composed of the particles and heavy metals and organic molecules. It should be also taken into account that organic molecules can enhance their neurotoxic features during an interaction with specific environmental factors (heating, oxidation, etc.).

In modern agriculture, accurate material delivery to the plants for precision farming techniques, enhancement of plant capability to absorb nutrients, disease detection and control/withstanding environmental pressure are performed using nanoparticles (Singh et al., 2015; Duhan et al., 2017). Nanoencapsulated conventional fertilizers, pesticides and herbicides provide precise dosage release of nutrients and agrochemicals to the plants. In this context, implementation of nanotechnologies in precision agriculture needs to be accompanied by deep understanding of nanoparticle absorption and dust-forming capability, biodegradation process and consequent neurosafety level.

It can be also expected that the particles originated from highly polluted regions can be enriched with local neurotoxicants and spread them worldwide. During traveling, such particles can broadly deliver local neurotoxicants to the human brain. Absorption of hydrophobic neurotoxicant, for instance polychlorinated biphenyls, at the particle surface can make them hydrophobic and so especially “invisible” to the BBB.

Therefore, a special threat to the CNS is associated with absorption capability of the particles to bind different neurotoxic, environmental pollutants. Micro- and nano-sized particles can serve occasionally as the composite carriers for the delivery of specific neurotoxicants to the CNS due to ability of the particles to overcome the BBB.

## Planetary Micro- and Nano-Sized Particle Effects on the CNS

Recently, new effects of long-duration spaceflight on brain anatomical configuration, i.e., a narrowing of the central sulcus and upward shift of the brain, and a narrowing of the cerebrospinal fluid spaces at the vertex were found in astronauts (Roberts et al., 2017). In laboratory-based model experiments, it was shown that hypergravity changed glutamate and GABA transporter functioning in presynaptic nerve terminals (Borisova and Himmelreich, 2005). Exposure of astronauts to planetary micro- and nano-sized dust particles during manned extraterrestrial missions that include extravehicular activities can significantly aggravate morphological and physiological effects found in brain structure and functioning.

Solid dust particles are in the planet-forming regions and at the surfaces of small solar-system bodies, however, their role is not clear yet (Blum, 2010). It was shown recently that dust particles in the interstellar medium were predominantly formed by carbon. Meteorites are abundant with carbon, the Murchison and Allende chondrites contain up to five parts per million carbon enriched in carbon-13 (Garai et al., 2006; Pizzarello and Shock, 2010). Organic carbon analysis from the Tissint Martian meteorite demonstrated the past existence of subsurface organic-bearing fluids on Mars (Lin Y. et al., 2014). Interstellar diamonds coexisted with highly reduced carbides of Si, Mo, W, and Ti (Jones et al., 2004; Garai et al., 2006). Microdiamonds were quite elusive in the interstellar space and can be detectable when consisted of more than 10% of carbon (Lewis et al., 1987). However, formation of diamonds in interstellar environments is still debated (Garai et al., 2006).

Halite crystals hosted in meteorites contained precursor and intermediate organic compounds that could make up amino acids. The organic matter included a mixture of C-, O-, and N-bearing macromolecular carbon materials and aromatic, ketone, imine, and/or imidazole compounds (Chan et al., 2018). As different types of organic molecules were hosted in meteorite’s halite crystals (Chan et al., 2018), planetary dust particles can be a brain delivery cargo for these organic molecules. The presence of potentially neurotoxic organic components at planetary dust particles can aggravate the neurotoxic features of the latter.

It is restricted amount of experimental data regarding health effects of planetary micro- and nano-sized dust particles. The particles of Lunar dust adhered to space suits and were transported into spacecrafts (Wallace et al., 2009; Rehders et al., 2011). Harmful Lunar dust effects on primary exposed tissues were shown in several studies (Lam et al., 2002; Rehders et al., 2011). Irritation of the eyes, the respiratory system and skin resulted from direct contact of Lunar dust with the human body was demonstrated during several Apollo missions. Lunar and Martian dust simulants caused preferential damage of the alveolar suppressor macrophage subpopulation and a dose-dependent increase in cytotoxicity (Latch et al., 2008). Comparative analysis of acute effects of dust particles in the lung indicated that Lunar dust was more toxic than TiO_2_ particles and Martian dust effect was comparable to that of quartz (Lam et al., 2002).

Effects of planetary dust particles on secondary exposed tissues, such as nervous system, are almost unknown. It was shown in the experiments *in vitro* that there was an increase in L-[^14^C]glutamate binding to isolated rat brain nerve terminals in low [Na^+^] media and at low temperature in the presence of Lunar dust, whereas Martian dust caused significantly lesser changes under the same conditions. This exceptional ability to increase glutamate binding to the brain synaptosomes can be explained by specific shape of Lunar dust particles containing sharp angles. However, the exact mechanisms of this effect need to be further investigated. This feature of Lunar dust particles can affect the ambient glutamate level in the synaptic cleft and extracellular glutamate homeostasis in general. It was shown in fluorimetric experiments that potential of the nerve terminal plasma membrane and synaptic vesicle acidification remained unchanged in the presence of Lunar and Martian dust simulants (Pozdnyakova et al., 2017). Also, Lunar dust simulant induced the enhanced expression of inducible nitric oxide synthase in the murine macrophage cell line (Chatterjee et al., 2010). Exposure to Lunar dust and other micro- and nano-sized particles can provoke inflammation (Oberdörster et al., 1994; Chatterjee et al., 2010; Bourdon et al., 2012), which in turn can alter permeability of the BBB (Abbott, 2000). An increase in duration of Lunar and Martian simulant presence in the lung transformed the acute inflammatory response to a chronic inflammatory lesion (Lam et al., 2002).

As native Martian dust can contain carbon components, the latter were mixed with inorganic Martian dust simulant in ground-based model experiments. Upgraded carbon-containing Martian dust simulant can have deleterious effects on extracellular glutamate and GABA homeostasis in the CNS and glutamate- and GABA-ergic neurotransmission misbalancing excitation and inhibition (Pozdnyakova et al., 2017).

Components from which Lunar and Martian dust particles are composed of can be also neurotoxic in case of their conversion into the ionic form. For example, the main components of Lunar and Martian dust simulants are oxides of physiological trace metals ferrum and manganese (FeO, Fe_2_O_3_, and MnO), excess of which jeopardizes brain health (Borisova, 2014). Experimental data confirmed that dissolution of metal ions from metal oxide nanomaterials underscored their neurotoxicity (Karmakar et al., 2014).

In the planetary exosphere, in contrast to ground-based conditions, the physical and chemical properties of planetary micro- and nano-sized particles can be modified by several factors, such as radiation, low gravity, UV, etc. Toxic effects of native Lunar dust can significantly exceed those of simulants made from Earth materials. This is because of unique features of native Lunar dust that is forming under conditions of meteoroid impacts and extended radiation exposure in the absence of oxygen and humidity. Lunar dust samples acquired *in situ* exist in a pristine state and their surface reactive chemicals can be preserved and also Lunar dust region-to-region variability requires *in situ* analysis (Linnarsson et al., 2012). Thermal fluctuation and the low-altitude levitation of Lunar dust can aggravate it’s *in situ* effects (Rosenfeld et al., 2016). Health risks associated with dust inhalation increased in a microgravity environment due to reduction of gravity-induced sedimentation. Inhaled particles tended to deposit more peripherally beyond the mucociliary clearance system and thus can be reserved in the lungs for longer periods (Peterson et al., 2008; Darquenne, 2014).

Combination of prolonged particle-induced irritation, inflammation, stress, microgravity-associated brain effects, ultraviolet and radiation during long-duration space missions can aggravate neurological consequences from planetary dust exposure. Vice versa, harmful effects of planetary dust can worsen physiological anomalies associated with long-term extraterrestrial missions (Pozdnyakova et al., 2017). Express on-board assessment of neurotoxicity of planetary micro- and nano-sized particles as well as possible organic inclusions needs to be developed.

## Modification of Micro- and Nano-Sized Particle Neurosafety by Biocorona Formation

Micro- and nano-sized particles due to their small size and large surface-to-mass ratio can self-assemble with a wide variety of biomolecules, that is, proteins, lipids, nucleic acids, carbohydrate polymers, etc., thereby being coated with biocorona (Saptarshi et al., 2013). The latest plays a critical role in particle-cellular interactions, development of consequential biological effects, particle accumulation, degradation and clearance from organisms (Saptarshi et al., 2013). For instance, plasma proteins tend to associate with nanoparticles forming protein corona surface (Pederzoli et al., 2017). This surface can have a strong impact on particle biodistribution and influence their efficacy and toxicity. The protein corona consists of two poorly delimited layers, i.e., a hard corona layer formed by strongly associated proteins, and soft corona that represents an outer layer consisted of loosely bound proteins (Pederzoli et al., 2017). Plasma protein corona of magnetic nanoparticles can provoke toxic side effects because the initial cellular interaction leads to downstream modification of further interaction with cells and tissues (Lundqvist et al., 2011; Mahmoudi et al., 2011). Silver nanoparticles induced SH-SY5Y neuroblastoma cell mortality through fragmentation of DNA and spontaneously formed a static complex with tau protein via hydrogen bonds and van der Waals interactions inducing slight changes in the tau protein structure (Rahmani et al., 2017). Fascinatingly, protein biocorona-coated nanoparticles have natural physiological analog, i.e., ferritin, a complex of proteins and nanoparticles representing mineral crystallites resembling ferrihydrite (Borysov et al., 2014). Not all types of micro- and nano-sized particles can form biocorona. In general, particle-biomolecule associates are temporal complexes. Multidisciplinary approaches to acquire knowledge about particle biocorona features are urgently required for complete understanding of its impact on particle safety (Pederzoli et al., 2017) and it is a crucial necessity to recognize molecular mechanisms of biocorona formation (Saptarshi et al., 2013).

The particle size, composition, surface properties, e.g., hydrophobicity, presence of specific functional groups, pH and temperature, influence protein adsorption at the particle surface (Kirchner et al., 2005; Walling et al., 2009; Soenen et al., 2011, 2015). For example, polyacrylic acid gold nanoparticles (12 nm) bound fibrinogen more efficiently that those smaller in size (7 nm) (Deng et al., 2012). Silica nanoparticles (15 nm) caused more considerable changes in carbonic anhydrase I protein structure as compared to the smaller ones (6 nm) (Lundqvist et al., 2004). However, literature data on the dependence of biocorona formation from the particle size is contradictional. Silica nanoparticles of different size had identical plasma protein adsorption profiles (Dutta et al., 2007; Saptarshi et al., 2013). Particle shape and surface charge effects on biocorona formation are also uncertain. The titanium dioxide, silica dioxide and zinc oxide nanoparticles had similar surface charges in a buffer; however, they bound different plasma proteins (Deng Z.J. et al., 2009). Superparamagnetic iron oxide nanoparticles coated with the polyvinyl alcohol polymer, surface charge of which was negative and neutral, adsorbed more serum proteins than the dextran-coated ones. Polyvinyl alcohol polymer-coated nanoparticles showed higher blood circulation time than the dextran-coated ones (Sakulkhu et al., 2014). Protein binding was influenced by shape of titanium dioxide nanoparticles (nanorods and nanotubes). The nanoparticles with uncharged surfaces bound less amount of proteins than those with positive and negative charges (Deng Z.J. et al., 2009; Fertsch-Gapp et al., 2011; Deng et al., 2012).

Vice versa, structural, dynamical and functional properties of corona-forming biomolecules can be significantly modified when they became corona constituents (Shemetov et al., 2012; Saptarshi et al., 2013; Lin et al., 2017). Causing misfolding of proteins, the particles can contribute to the development of Alzheimer and Huntington diseases, type 2 diabetes and the dialysis-related amyloidosis (Linse et al., 2007). The nanoparticles of curved surface area can irreversibly affect protein secondary structure (Worrall et al., 2006). Drug bound-polysorbate 80 coated nanoparticles transported across the BBB that was assisted by apolipoproteins B/E and occurred most probably through receptor-mediated endocytosis (Kreuter et al., 2002). Making comparison between *in vitro* and *in vivo* experimental data, it is essential to consider biocorona formation process (Monopoli et al., 2011; Lin et al., 2017).

Interaction of micro- and nano-sized particles with biomolecules is dynamic process that is characterized by continual protein adsorption and desorption from the particles (Casals et al., 2010). Absorption of the plasma proteins by silica and polystyrene nanoparticles was augmented with an increase in the plasma protein concentrations (Monopoli et al., 2011; Saptarshi et al., 2013). Albumin and fibrinogen as more abundant proteins may initially bind to the particle surface and then can be subsequently replaced by other proteins that have higher binding affinity to the particle surface (Saptarshi et al., 2013).

Micro- and nano-sized particles can interact with natural organic matter of air, soil and water resources. The particles can absorb natural organic compounds originating from plant and animal residue decomposition, microbial products, etc. Environmental particles can be immobilization platform for different microorganisms, which in turn become more stable during this immobilization. Natural organic matter can modify nanoparticle toxicity (Verneuil et al., 2015; Zhang et al., 2015; Lin et al., 2017). Besides that, fungal spores, pollen, viruses, microorganisms (bacteria, archaea, algae, and fungi) and biological fragments can be considered *per se* as aerosol bioparticles. A typical size range of aerosol bioparticles varies between 0.05 and 0.15 μm for viruses, 0.1–4.0 μm for bacteria, 0.5–15.0 μm for fungal spores and 10–30 μm for pollen (Després et al., 2012). The concentration of aerosol bioparticles comprises approximately 25% of total global aerosol mass but varies in dependence on location and season (Jaenicke, 2005; Lang-Yona et al., 2012). Season biological particles can cause allergic disease (Fuzzi et al., 2015).

Therefore, neurosafety of the particles can be modified by biocorona formation at the particle surface from environmentally derived protein neurotoxins and different membrane- active and pore-forming substances. Also, it occurs during contact with organisms, where the way of particle delivery is crucial. In organisms, environment-derived neurotoxic particle biocorona can be replaced by abundant physiological proteins, thereby releasing neurotoxins. As biocorona-formation mechanisms are still remaining unclear in details, it is difficult to predict micro- and nano-sized particle neurosafety. From the one hand, biocorona formation depends on various particle properties, e.g., particle size, shape, surface characteristics and composition. From the other hand, a wide variety of biomolecules from which biocorona can be composed of makes difficult precise neurosafety prognosis. Particle biocorona composed of natural organic matter requires further investigation and characterization. Neurosafety prognosis should be developed for each combination of particle-biomolecule complex.

## Biocorona-Related Discrepancy in Particle Neurotoxicity Data

Currently, general principles and methods for assessment of micro- and nano-sized particle neurosafety are quite unclear. Multifactorial complexity of particle neurotoxicity analysis and diversity of the experimental data obtained using different methodological approaches leads to controversial conclusions regarding their neurosafety. We suggest that discrepancy of the results from *in vitro* experiments can be associated with variability of the systems, in which particle neurotoxicity was analyzed. For example, different prognosis regarding toxicity of carbon dots can be made from the experiments with tissue preparations, cell cultures and animal studies (Bhunia et al., 2013; Luo et al., 2013; Wang et al., 2013; Borisova et al., 2015; Havrdova et al., 2016). We suppose that this is because of spontaneous formation of the primary particle biocorona using protein components presenting in the incubation media of the cell cultures (as usual the cellular incubation media contain serum) and this process occurs before direct interaction of the particles with the cells. Exogenous proteins as components of the cell culture incubation media can significantly distort and even eliminate factual particle effects and this fact can mask their neurotoxic properties.

**FIGURE 3:**
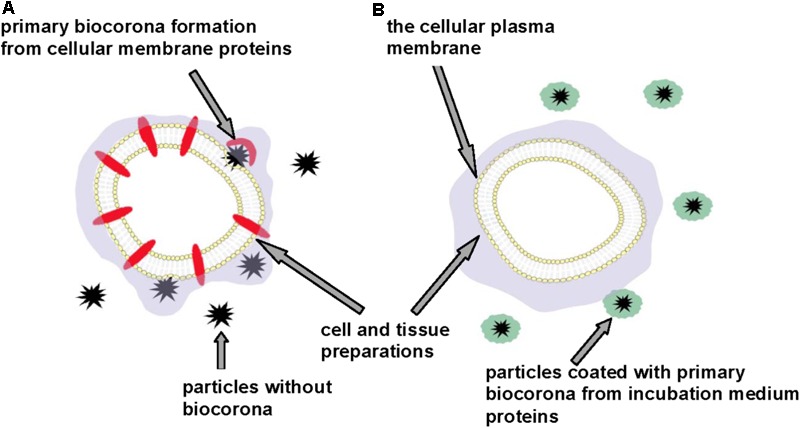
Discrepancy in neurotoxicity risk assessment of micro- and nano-sized particles. Cell and tissue preparations without **(A)** and in the presence of **(B)** additional protein components. Primary particle biocorona can be formed from functionally important proteins located in the cellular plasma membrane **(A)** or from incubation medium proteins thereby making particles inert regarding interaction with the plasma membrane and masking their neurotoxic properties **(B)**.

We suppose that exact acute effects of micro- and nano-sized particles on the cellular plasma membrane and in general particle-cellular interaction can be accurately monitored using different cell and tissue preparations, the incubation media of which do not contain additional protein components (**Figure 3**). This approach can prevent masking effects of the primary biocorona consisted of incubation medium protein components that cover authentic neurotoxic potential of the particles. Factual membrane activity of the particles and their capability to form biocorona directly from plasma membrane proteins (and lipids) can be analyzed using this approach. We suggest that neurotoxicity of carbon dots, nanodiamonds, maghemite nanoparticles, nanocrystals shown in the experiments with the nerve terminal preparations (Borisova et al., 2015, 2017; Pozdnyakova et al., 2016; Horák et al., 2017; Sojka et al., 2017) can be associated with spontaneous formation of the primary particle biocorona from functionally important synaptic proteins located in the plasma membrane.

Comparative analysis of neurotoxicity of different types on micro- and nano-sized particles in order to have less toxic ones or evaluation of the particle size, shape and surface effects can be properly made using identical *in vitro* system.

During intranasal entry, the primary environment-derived biocorona of the particle surface can be spontaneously replaced by the secondary one consisted of mucosa proteins, and in case of particle access to the circulation the secondary biocorona can be formed from serum proteins. The important question is to what extent existed environmentally-derived particle biocorona can be replaced by the physiological one in organism. How easy neurotoxic substances and heavy metals absorbed at the particle surface can be released in organisms and whether or not biocorona changes the particle distribution and clearance in organism.

## Conclusion

**FIGURE 4:**
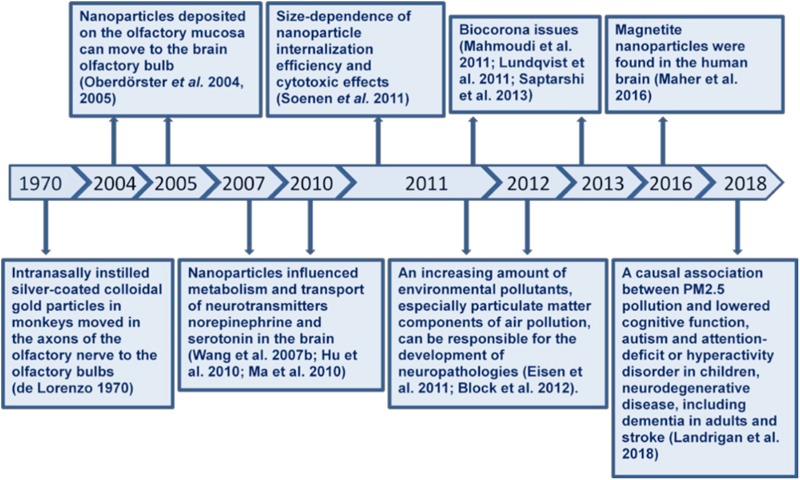
A chronologic timeline diagram of important milestones.

Environmental, engineered and planetary micro- and nano-sized particles can reach the CNS during inhalation due to their unique ability to bypass the BBB, affect neuronal functioning and provoke development of neuropathologies. An airborne pollution particulate matter disperses globally and crosses state boundaries, oceans and continents, thereby significantly widening the negative health consequences and enhancing the importance of its neurotoxicity risk assessment. Moreover, heavy metals and various neurotoxic substances from environment can be absorbed at the particle surface and occasionally use them as particulate carriers for brain access. The particles originated from highly polluted regions can be enriched with local neurotoxicants, thereby not only spreading them worldwide, but also being simultaneously brain delivery composite carriers during their traveling. Spontaneous particle-biomolecule interactions in organism and environment, and so biocorona formation can modify particle features and their distribution and clearance from organism. Particle-biomolecule interaction mechanisms are far from being clear and can be clarified by separate analysis of each type of these temporal complexes. **Figure 4** represents a timeline diagram of important milestones.

A common unified methodology for assessment of neurosafety of environmental, engineered and space micro- and nano-sized particles still remains almost undeveloped thereby making difficult predictive modeling. Prognosis of particle neurosafety potential is complicated because precursor materials, from which the particles are synthesized from, have different properties as compared to those in the micro- and nano-sized forms. Experimental data on particle neurotoxicity varied in dependence on the systems, where it was analyzed. Exogenously added protein components during experimental procedures can significantly mask factual particle effects. The direct acute effects of the particles on the cellular plasma membrane can be uncovered using preparations, the incubation media of which do not contain exogenously added protein components.

Neurosafety level of micro- and nano-sized particles depends on their composition, size, shape, surface properties, stability in organism and environment, capability to absorb neurotoxic substances, aggregate, form dust, and interrelate with different biomolecules. Newly arising changes in one of these parameters can break their initial authentic neurosafety.

## Author Contributions

The author confirms being the sole contributor of this work and approved it for publication.

## Conflict of Interest Statement

The author declares that the research was conducted in the absence of any commercial or financial relationships that could be construed as a potential conflict of interest. The reviewer MS and handling Editor declared their shared affiliation.
